# Sterculic Acid: The Mechanisms of Action beyond Stearoyl-CoA Desaturase Inhibition and Therapeutic Opportunities in Human Diseases

**DOI:** 10.3390/cells9010140

**Published:** 2020-01-07

**Authors:** Rafael Peláez, Ana Pariente, Álvaro Pérez-Sala, Ignacio M. Larráyoz

**Affiliations:** Biomarkers and Molecular Signaling Group, Neurodegeneration Area, Center for Biomedical Research of La Rioja (CIBIR), Piqueras 98, 26006 Logroño, Spain; rpelaez@riojasalud.es (R.P.); apariente@riojasalud.es (A.P.); aperez@riojasalud.es (Á.P.-S.)

**Keywords:** sterculic acid, inflammation, cell death, macular degeneration, metabolism

## Abstract

In many tissues, stearoyl-CoA desaturase 1 (SCD1) catalyzes the biosynthesis of monounsaturated fatty acids (MUFAS), (i.e., palmitoleate and oleate) from their saturated fatty acid (SFA) precursors (i.e., palmitate and stearate), influencing cellular membrane physiology and signaling, leading to broad effects on human physiology. In addition to its predominant role in lipid metabolism and body weight control, SCD1 has emerged recently as a potential new target for the treatment for various diseases, such as nonalcoholic steatohepatitis, Alzheimer’s disease, cancer, and skin disorders. Sterculic acid (SA) is a cyclopropene fatty acid originally found in the seeds of the plant *Sterculia foetida* with numerous biological activities. On the one hand, its ability to inhibit stearoyl-CoA desaturase (SCD) allows its use as a coadjuvant of several pathologies where this enzyme has been associated. On the other hand, additional effects independently of its SCD inhibitory properties, involve anti-inflammatory and protective roles in retinal diseases such as age-related macular degeneration (AMD). This review aims to summarize the mechanisms by which SA exerts its actions and to highlight the emerging areas where this natural compound may be of help for the development of new therapies for human diseases.

## 1. Introduction

Under normal conditions, lipogenesis and lipolysis coexist in dynamic equilibrium. Signals coming from the central nervous system as well as peripheral tissues determine the balance of synthesis and breakdown of triglycerides. Fat is mainly accumulated in adipose tissue, especially white adipose tissue, which is the principal energy storage organ [1,2]. There are two different sources of lipogenesis, de novo lipogenesis and circulating triglycerides. In tissues with high metabolic rate, such as the liver, or in adipose tissue, de novo lipogenesis is more active, although every single cell is able to perform lipogenesis [2]. In particular, human adipose tissue seems to be the principal tissue where de novo lipogenesis is carried out [3]. This type of lipogenesis is characterized by the transformation of carbohydrates into fatty acids, which are then esterified and stored as triglycerides if there is no demand of energy in the body. This process starts with the glycolysis of dietary carbohydrates in order to obtain acetyl-CoA. The enzyme acetyl-CoA carboxylase 1 (ACC1) converts acetyl-CoA into malonyl-CoA, which is next transformed to palmitate by the fatty acid synthase (FASN) [2]. Finally, the last step of de novo lipogenesis is carried out by stearoyl-CoA desaturase (SCD), the first rate-limiting enzyme involved in SFA desaturation [4]. SCD introduces a single double bound in palmitoyl-CoA to transform it into palmitoleoyl-CoA, in a reaction in which reduced nicotinamide adenine dinucleotide (NADH), flavoprotein cytochrome β5 reductase, as well as the electron acceptor cytochrome β5 are also involved [5]. In addition to palmitic acid, stearic acid is also one of the main substrates of SCD, which is desaturated and converted into oleic acid [6]. To a lesser extent, SCD also catalyzes the conversion of myristic acid into myristoleic acid [7].

**Figure 1:**
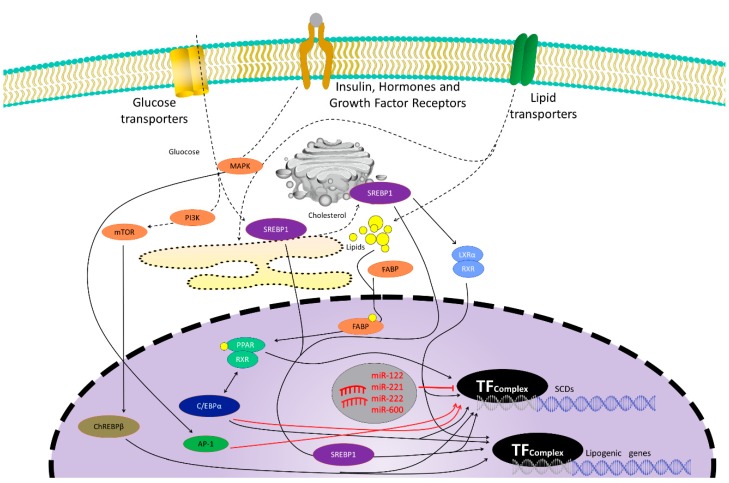
Genetic control of the stearoyl-CoA desaturase (SCD) family and other lipogenic gene expression. Promoter regions of SCD genes present many transcription factor binding sites, such as sterol regulatory element binding protein 1 (SREBP1), carbohydrate response element binding protein (ChREBP), CCAAT/enhancer-binding protein (C/EBP), liver X receptor (LXR), or peroxisome proliferator-activated receptor (PPAR). Black arrows represent inductive signals while red lines represent repressive signals that negatively modulate SCD genes expression. Glucose uptake or insulin signaling, but also lipid uptake, hormones, or growth factors binding to their receptors, signaling pathways such as phosphatidylinositol 3-kinase (PI3K), mitogen-activated protein kinase (MAPK), mammalian target of rapamycin (mTOR), or endoplasmic reticulum (ER) stress, promote direct or indirect transcription factor binding to SCDs promoters to modulate SCD gene expression. Some microRNAs (mirRNA121, 221, 222, and 600) have been reported to downregulate SCDs expression. Activator protein 1 (AP-1) is a transcription factor that reduces SCD1 expression after leptin stimulation. C/EBP induction can also downregulate SCD1 expression after bacterial infection.

De novo lipogenesis is stimulated when glucose and insulin blood levels are elevated [2] as they induce the activation of ChREBP (carbohydrate response element binding protein), SREBP-1c (sterol regulatory element binding protein 1c), and LXR (liver X receptor), specific transcriptional factors whose activation promotes lipogenesis [4,8] (Figure 1). Thus, SCD-1 hepatic expression is induced after a carbohydrate-rich intake through a SREBP-1c-dependent mechanism that involves the binding of LXR to a LXR-response in the SCD-1 promoter element through the activation of SREBP-1c transcription, but also through a SREBP-1c-independent pathway [8,9,10,11,12].

Sterculic acid (SA) is a cyclopropene fatty acid with numerous biological activities. In this review we describe how its ability to inhibit stearoyl-CoA desaturase (SCD) directly, can be of interest as a coadjuvant for the treatment for various diseases, such as, nonalcoholic steatohepatitis, Alzheimer’s disease, cancer, and retinal disorders. In addition, it displays anti-inflammatory properties, independently of SCD inhibition, which can be useful to treat other pathologies such as age-related macular degeneration (AMD).

## 2. Stearoyl-CoA Desaturase (SCD)

Stearoyl-CoA desaturase (SCD) is an enzyme which is known to be active in the conversion of saturated fats into (MUFAs). While mouse genome presents 4 isoforms (SCD1-4), human genome only has two isoforms (SCD1 and SCD5). In humans, both enzymes have the same catalytic activity and generate the same products, but they present different locations and effects [13]. SCD1 is located in the ER of cells in many tissues (lung, pancreas, skeletal muscle, brain, adipose tissue) while SCD5 is only located in brain and pancreas [14,15,16]. Although a compensatory effect was observed in some breast cancer models, SCD5 [8] is not able to restore the effects of SCD1 deficiency [17]. Recently, it has been described that SCD5 is also expressed in the fetal brain of dogs, bovines, and birds [18]. SCD5 promotes cell differentiation of melanoma cells and its expression is associated with less malignant tumors. Its expression is downregulated during tumor progression and related to the epithelial–mesenchymal transition reverted process [13]. SCD5 expression is associated with the reduction of intracellular pH, vesicle movement, and pro-tumoral protein secretion [19]. It has also been described that miR-221 and miR-222 have a negative feedback connection to SCD5 expression [13]. Human SCD5 expression in mouse neuronal cells alters lipids ratio and controls the differentiation stage of the cells, as well as it promotes cell proliferation through downregulation of EGF/Akt/Erk and Wnt canonical signaling pathways, while noncanonical pathway of Wnt and its ligands are increased [18].

**Figure 2:**
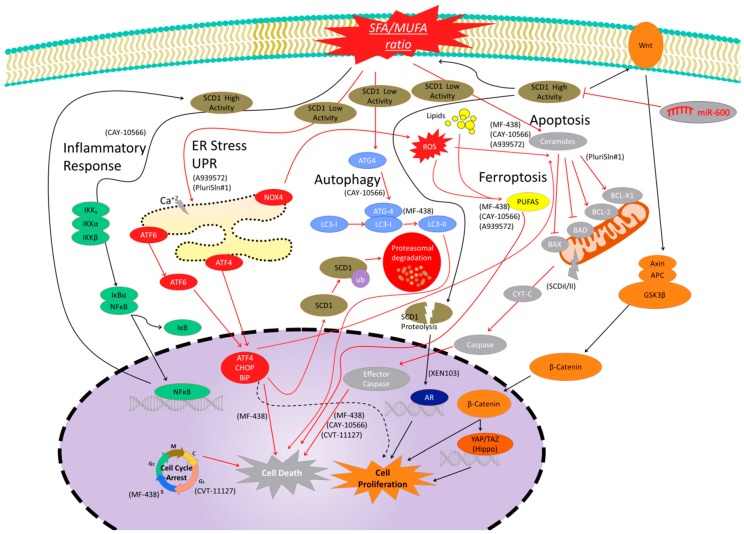
Signaling pathways modulated by SCD1 activity and levels. Black arrows are signals of cell proliferation or associated with high SCD1 expression or activity, while red arrows are signals linked to cell death or decreased SCD1 expression or activity. Inflammatory pathway is shown in green. SCD1 inhibition reduces the pro-inflammatory environment and nuclear factor kappa-B (NFκB) signaling to reduce cell proliferation, while NFκB signaling activation promotes transcription factors binding to SCD1 promotor and gene expression. The Wnt/β-catenin pathway is related to cell proliferation and it is shown in orange. High SCD1 levels are linked to increased β-catenin signaling and increased levels of wingless-related integration site (Wnt) ligands to induce cell proliferation. Hippo pathway is a β-catenin-related signal cascade, which is associated with cell proliferation, and SCD1 inhibition has been demonstrated to be associated with decreased levels of Hippo target genes. This pathway is shown in dark orange. The SCD1 peptides from proteolytic controlled degradation activate androgen receptor (AR) signaling to promote cell proliferation. This pathway is shown in dark blue. The autophagy cell death pathway is a protein cascade which is upregulated after SCD1 inhibition. Some central elements of this pathway are shown in light blue. The endoplasmic reticulum (ER) stress signal pathway is shown in red. This cascade is linked to misfolded and unfolded proteins to induced cell death. SCD1 inhibition has been linked to the upregulation of the elements of the ER pathway to promote cell death. SCD1 is also ubiquitinated by this pathway to promote protein degradation. However, light ER pathway activation has also been related to cell survival (dashed black arrows). Apoptosis is a programmed cell death shown in grey that is activated after SCD1 inhibitor treatments. Increased ceramides, mitochondrial effector pathway, caspases, and other pathway effectors have been detected after treatments. Ferroptosis is another cell death mechanism (yellow) that has been shown to be activated after SCD1 Inhibition. Finally, SCD1 inhibition has been demonstrated to induce cell cycle arrest in different checkpoints to promote cell death. Molecules in parentheses are SCD1 inhibitors used in the literature to elucidate the SCD1-related pathways.

SCD1 transforms stearic or palmitic acids to oleic and palmitoleic (MUFAs) [20]. MUFAs are the main contributor to total membrane phospholipids and cholesterol esters. Pharmacological inhibition of SCD1 reduces lipid and MUFAs synthesis, suggesting that SCD1 is a master regulator of membrane composition [21]. This fact points out that SCD1 is a central element of lipid metabolism and body weight control [14]. Interestingly, its deficiency is also associated with a narrow eye fissure [22] and skin abnormalities [17]. Recently, it has been demonstrated that SCD1 protects against the palmitate-induced cytotoxic effect in mesenchymal stromal cells and osteoblasts [23]. SCD1 modifies the SFA/MUFA ratio through its catalytic activity and induces caspase 3/ activation, endoplasmic reticulum (ER) stress, and inflammation [23] (Figure 2). SCD1 overexpression is mediated by LXRs, peroxisome proliferator-activated receptors (PPARs), sterol regulatory element-binding proteins (SREBPs), CCAAT/enhancer-binding proteins (C/EBP)α, etc. [8,23,24]. Curiously, C/EBP also downregulates SCD1 expression in liver and udder during *E. coli* mastitis and is associated with a SREBP-1 downregulated expression [25].

SREBP1 is the master regulator in fatty acid metabolism, and its activation has been linked to obesity, fatty liver disease, insulin resistance, autoimmune diseases, as well as cancer development. SREBP1 controls SCD1 expression [21,26], and therefore factors activating SREBP1 signaling, such as carbohydrate intake, activate SCD1 as expected [27]. LXRs are other factors that control SCD1 expression. LXRα disruption in mice is associated with reduced levels of SCD1 and altered lipogenesis [28], whereas LXR agonist activation promotes its expression [18,29]. Other negative regulators, such as miR-122, reduce the expression of SCD1 and as a consequence, many other genes associated to lipid metabolism [30]. Recently, it has been observed that miR-600 inhibition increases SCD1 expression and oleic acid levels. Consequently, Wnt ligands increase, as well as β-catenin transactivator activity, to promote cell proliferation [31]. Recently, it has been described that SCD1 expression is also controlled by ER stress signaling in retinal pigment epithelial cell lines. In this sense, overexpression of elements of this pathway or pharmacological induction decrease SCD1 expression, as well as cell proliferation or insulin resistance. ER stress activates ubiquitination and proteasomal mechanisms leading to degradation of SCD1 [32]. Glucose, insulin, but also growth factors, or lipids activate SCD1 expression using different signal pathways [8,33]. For example, phosphatidylinositol-3 kinase (PI3K) and mammalian target of rapamycin (mTOR) are activated in response to insulin [34]. Mitogen-activated protein kinase (MAPK) signaling has been demonstrated to activate SCD expression in an epidermal growth factor receptor (EGFR) dependent manner [18,35], although this pathway also represses the gene expression by activator protein 1(AP-1) after leptin stimulation [36]. Metformin, an antidiabetic drug, represses SCD1 expression by 5’adenosin monophosphate-activated protein kinase (AMPK) signal and the reduction of SREBP-1c expression [37].

Many SCD1 inhibitors have been tested with good results in liver diseases such as nonalcoholic fatty liver, diabetes, dyslipidemic failure, and hepatitis C virus (HCV) infections [17]. For example, MK-8245 is right now in advanced phase 2 human clinical studies [14] (Table 1).

### 2.1. Cancer

SCDs are overexpressed in many cancer types [14,59,60] and SCD1 expression is associated with the reduction of relapse-free survival of patients of breast cancer [61]. SCD1 activity increases MUFAs levels in cell membrane to promote cell viability [21]. For example, SCD1 is overexpressed in samples of patients with anaplastic thyroid carcinoma (ATC) and well-differentiated thyroid carcinomas, and its expression correlates with tumor aggressiveness and poor prognosis in human hepatocellular carcinoma (HCC) patients [14,40,62]. In prostate cancer cells, SCD1 proteolytic cleavage generates a small peptide that activates androgen receptor (AR) signaling to promote cell proliferation [63] (Figure 2). It has also been described that SCD1 can induce the formation of Wnt ligands to promote β-catenin signaling [31]. SCD1 knockout mice are viable but present skin abnormalities and weight reduction [64] suggesting that cancer treatments based on SCD inhibition should be restricted to a local and short action. Furthermore, SCD1 silencing reduces the survival of prostate cancer cell lines [65] and cell proliferation and tumor in lung cancer and animal models [48,66] reinforcing the idea that SCD can be a novel therapeutic target against some tumors.

Cancer stem cells (CSCs) are usually associated with chemoresistance and cancer. High levels of SCD1 have also been observed in CSCs [17,31,45,67,68]. In lung tumor spheroids, SCD1 expression was significantly upregulated [67] and SCD1 inhibition with MF-438 reverts tumoral cisplatin resistance [45,69]. Pharmacologic inhibition of SCD1 induces a selective cell death of cells that express the CSCs marker aldehyde dehydrogenase 1-A1 (ALDH1A1) with a 100-fold selectivity over their equivalent normal cells [41,67]. Tesfay and coworkers have described that chemically, or genetically, SCD1 inhibition activates apoptosis and ferroptosis in ovarian cancer cells. SCD1 inhibition decreases the monounsaturated/saturated lipid ratio to increase proapoptotic ceramides and to increase lipid oxidation linked to ferroptosis, ER stress, and apoptosis [17,32]. Different signaling pathways, such as Hippo or Wnt, are regulated by lipid metabolism enzymes such as SCD1 [31]. Other authors have observed that SCD1 inhibition (through SCD siRNA) affects the cardiolipin levels, promoting cytochrome C release and apoptotic cell death in breast and prostate cancer cells [47]. Furthermore, SCD1 silencing increases ER and oxidative stress, SFA accumulation in cell membranes, and promotes unfolded protein response (UPR) activation [21]. SCD1 inhibition is linked to a β-catenin inactivation and the reduction of the nuclear localization and signaling of yes-associated protein/transcriptional coactivator with PDZ-binding motif (YAP/TAZ) [68,69]. YAP/TAZ are onco-proteins of Hippo signaling observed in multiple tumor types and CSCs [70], whose target genes are repressed by SCD1 inhibition [68]. Other signaling pathways such as nuclear factor-kB (NF-kB), Hedgehog, and Notch are also associated with SCD1 in ovarian, unfolded protein response (UPR) in liver and EGFR in lung CSCs [31]. NF-kB is downregulated after SCD1 inhibition with CAY10566, while p65 overexpression increases SCD1 levels in ovarian CSCs to generate a positive feedback and promote tumor cell proliferation [42,71]. A low dose of SCD1 inhibitors in combination with ferroptosis inducers could be a possible option to treat tumors. Furthermore, cancer stem cells are highly susceptible to this combined treatment, and therefore this could be a good option for the treatment of resistant and recurrent ovarian tumors [17].

Small-molecule inhibitors MF-438, CAY10566, and SC-26196 reduce sphere-forming efficiency of lung and ovarian CSCs, with anoikis activation and cellular damage [31]. Inhibition of SCD1 by A939572 inhibits growth in lung tumor cells and reduces the tumor volume in mice bearing human gastric cancer xenograft [14,39]. CVT-11137 is a SCD inhibitor that promotes G1 cycle arrest and programmed cell death in lung cancer cells [48]. A939572 is also effective in growth inhibition of clear cell renal cell carcinoma (ccRCC) [40] and, when it is combined with a mTOR inhibitor, abrogates tumor growth in in vitro and in vivo models [38]. A939572 and MF-438 decreased cellular proliferation in many anaplastic thyroid carcinoma cell lines, although MF-438 was not effective in tumor reduction of xenograft mouse model [14]. CAY-10566 is another SCD1 inhibitor that reduced cell viability by apoptosis induction in human HCC cells [43], and also in colon cancer cells xenografts in mice [44]. Autophagy activity has also been observed in human HCC cells after CAY-10566 treatment, although other inhibitors do not have this effect [14].

### 2.2. Dermatology

Sebaceous gland atrophy and other skin disorders have been reported in SCD knockout mice [72] and also after SCD1 inhibition by A-939572 administration [49], possibly mediated through blockade of the androgen-induced transcription of SCD1 gene [49]. Therefore, SCD1 inhibitors have been proposed for the treatment of acne [14].

### 2.3. Alzheimer’s Disease

Memory decline is the hallmark of Alzheimer’s disease (AD). Loss of synaptic contacts in the cerebral cortex and hippocampus, caused in part by cytoskeleton disruption, provokes memory failure. Our group has previously reported that adrenomedullin has a crucial role in this process [73,74]. Interestingly, SA is able to reduce adrenomedullin expression (AP, RP, APS, IML, in preparation). Furthermore, brain samples of AD patients present higher levels of MUFAs in the mid-frontal cortex, temporal cortex, and hippocampus. In these patients, SCD1 and SCD5 mRNA levels were also elevated more than that of the control patients [75]. Other authors have reported the association of SCD1 overexpression in neuronal H4 cells and an increased secretion of amyloid beta 42 [14], suggesting that SCD can also represent a potential target in AD therapy.

### 2.4. Liver

Nonalcoholic fatty liver disease (NAFLD) is characterized by lipid accumulation in the liver, and it is often associated with obesity, insulin resistance, hypertension, and dyslipidemia [76]. Up to 40% of NAFLD patients progress to the more advanced form of the disease, nonalcoholic steatohepatitis (NASH). Currently, SCD1 inhibitors are under investigation for NASH treatment [27]. MK-8245, an SCD inhibitor, has shown antidiabetic and antidyslipidemic efficacy in preclinical animal models. Hepatic steatosis, hepatocellular degeneration, and inflammatory cell infiltration were also ameliorated after the treatment [14].

SCD1 inhibition can also be a novel therapeutic strategy for the treatment of HCV infection and reduce viral replication in human hepatoma cells [14,77]. Mechanistic investigations suggested that SCD1 inhibition disrupts the integrity of membranous HCV replication complexes and makes HCV RNA susceptible to nuclease mediated degradation [14].

### 2.5. Atherosclerosis

Foam cell formation and lipid accumulation, are the first steps of atherosclerosis and many of these foam cells are derived from vascular smooth muscle cells (VSMCs) [78]. The role of SCD1 in the atherogenic inflammation is controverted. While SCD1-deficient mice show important atherogenic lesions, its silencing reduces the plaques in wild type animals subjected to a high-cholesterol diet [72,79]. Interestingly, oxidized low-density lipoproteins reduce SCD1 expression and their signaling, while SCD1 overexpression reduces VSMC foam formation in a lipophagy-mediated manner to reduce lipid droplets and atherosclerotic lesions. It has been demonstrated that SCD1 is associated with the suppression of inflammatory responses and increases the cholesterol reverse transport [72,80] promoting ABCA1 expression and transcription factor EB (TFEB) signaling [81].

### 2.6. Adverse Effects of SCD1 Treatments

It is important to note that SCD inhibition can also produce unwanted effects in the organism. Tissues where lipogenic mechanisms must be active to function normally can be affected by long systemic treatments with SCD1 inhibitors. For example, SCD1 knockout mice present skin and weight abnormalities with elevated levels of retinol and retinoic induced genes [64]. These animals present epidermal lipid barrier dysfunction with a subsequent thermoregulation failure, transepidermal water loss, and metabolic problems [82]. SCD1-deficient mice also present aortic atherosclerosis and an increased macrophage inflammatory response [83]. SCD1 inhibitor treatments usually cause sebocyte atrophy, hair loss, and eye dryness [84]. It has also been observed that SCD1 inhibitors abrogate the protective effects of SCD1 on palmitate-induced lipoapoptotic cell death of β-pancreatic cells [85] Furthermore, different studies in breast cancer models have demonstrated an upregulation of SCD5 in human cancer cells [8]. Recently, alternative FA desaturation pathways have been found in tumor cells. It has been observed that SCD inhibition upregulates sapienate levels in non-sensitive SCD1 inhibitor tumor cells such as liver carcinoma. Moreover, fatty acid desaturase 2 (FADS2), and its product sapienate, are also upregulated in lung and liver cancer tissues [86]. On the basis of these findings, the development of new SCD inhibitors with fewer adverse effects, as well as a better understanding of the mechanisms involved, are of crucial interest.

## 3. Sterculic Acid

SA is a cyclopropenoid fatty acid mainly obtained from the seeds of *Sterculia foetida* and represents more than 50% of its oil composition. SA is well-known because of the inhibitory effect it exerts on the enzyme SCD1, also known as Δ9-desaturase, both in vivo and in vitro [50,52,53,55,87,88,89]. This inhibitory effect has been proposed to be, in part, due to its highly strained and reactive propene ring and the presence of a double bond between C9 and C10 in its chemical structure [88]. Studies in 3T3-L1 adipocyte cells have shown that this inhibition occurs by downregulation of the enzyme activity without affecting the mRNA or protein levels of SCD [88,90]. Moreover, SA does not change transcription or translation levels of SCD in *Toxoplasma gondii* [55]. Thus, this inhibition could result from the irreversible binding of the sulfhydryl groups of the enzyme with cyclopropene groups [91] or through the conversion of SA into sterculoyl-CoA, which seems to be its activated form [92]. However, Dallaire et al. found in mammary cow tissue a 31% increase of SCD-1 mRNA levels, after sterculic oil (SO) administration for four days. The authors explained that this was a consequence of a decrease in the levels of SCD products, which leads to an increase of SCD-1 transcription in order to counteract this deficiency [93].

SA is commonly administrated as part of SO [50,52,53,93,94,95]. SO not only contains SA, which is its major component, but also malvalic acid, another cyclopropene acid with similar properties to that of SA, including the SCD-inhibitory capacity [96,97,98,99]. Beneficial effects of SO include improvement of glucose tolerance and blood pressure, reduction of body mass, and an amelioration in the serum levels of triglycerides and adiponectin, among others [51,52,53,88]. However, several side effects have also been described. These include hypercholesterolemia in hamsters, problems in hen reproduction, carcinogenesis, and inhibition of the anticarcinogenic effect of conjugated linoleic acid (CLA) in rats due to SCD activity disruption [90,94,100,101]. In particular, SA has been shown to have a potent luteolytic effect in ovines by inhibition in the synthesis of progesterone, which causes luteal regression [102].

No hepatotoxicity was observed in hamsters fed with and SO-enriched diet although a 20-fold plasma alanine transaminase (ALT) increase was detected when animals were fed with both SO- and cholesterol-enriched diets. This was proposed to be mediated by the accumulation of potentially toxic derivatives of cholesterol such as hydroxyl- or epoxy-cholesterol [94]. ALT plasma concentration was also under hepatotoxic levels in Otsuka Long–Evans Tokushima Fatty rats treated with SO [52]. Moreover, no effect of SCD1 inhibition using 50 to 100 μM SA was found on the proliferation of preadipocyte differentiation or viability of differentiated adipocytes [87].

Several studies have been done in animal models to determine the effects of this natural cyclopropene in milk composition and production [50,89,93,103,104,105]. It has been especially studied in cows [93,103,104,105], where SA, which was administrated as an abomasal infusion of SO, resulted in a reduction of milk yield in long-term periods [93] but had no effect as compared with the controls when it was infused in short-term periods [103,104]. Moreover, an increase of milk fat yield was observed [93,103] although these differences were compensated with a lower total milk yield in the case of long-term experiments [93]. In these experiments, an inhibition of 70% to 83% of SCD1 activity was found [93,103,104], and consequently, milk MUFA ratios of stearic, oleic, and palmitoleic acid, as well as other SCD1 products, were significantly reduced with respect to their saturated form [93,103,104,105]. Something similar has been described in lactating ewes after jugular SA infusion, where a reduction of milk MUFA content was found without affecting milk yield [89]. This has been corroborated in vitro in bovine adipocytes, where SA decreased the percentage of MUFA in these cells while the total content in fatty acid remained stable [87]. Studies in hamsters fed with an SO-enriched diet showed an important decrease in the liver and adipose tissue content of palmitoleic and oleic acid content and a small increase in hepatic linoleic acid, which was suggested as an adaptive response for the maintenance of membrane fluidity given the shortage of MUFA [94]. Adipose storage tissue or body weight did not change in these experiments. However, a decrease in body weight was reported when SO was administrated together with cholesterol [94].

### 3.1. Effects of SA on mRNA and Protein Expression

The changes elicited by SA administration on milk production provide useful information about the effect of SCD1, and consequently, SA on lipogenesis. The mRNA levels of several proteins involved in lipid metabolism have been measured in bovine adipocytes treated with SA in order to inhibit SCD1 enzyme activity [87]. Data showed a decrease in the expression of ACC involved in lipogenesis, as well as an increase in the levels of lipase E (LIPE), a gene related to lipolysis, which highlights the importance of SCD1 in lipogenesis [87]. In hamsters, SO had a small effect on hepatic mRNA levels, but when administrated together with cholesterol, ACC, as well as FAS (Fatty acid synthase), SREBP1a, and SREBP1c hepatic mRNA levels were also decreased [94]. In Otsuka Long–Evans Tokushima Fatty rats, FAS and SREBP1c mRNA levels were also reduced when SO was administered [52]. Levels of CPT1 (carnitine palmitoyltransferase 1), a gene of the fatty acid oxidation, were also increased in bovine adipocytes. The authors suggested a decrease of malonyl-CoA, which is a repressor of CPT1, as a consequence of a reduction in the levels of ACC that would provoke the increase of the mitochondrial fatty acid oxidation. Other genes reported to be increased by SA were the elongation of a very long-chain fatty acids protein 6 (ELOVL6) and glicerol-3-phosphate dehydrogenase (GPDH) [87].

Conversely, low density lipoprotein receptor (LDLR) and SREBP2 mRNA levels remained unchangeable in hamsters fed with a SO-enriched diet [94]. A decrease in the content of hepatic cholesteryl esters showed that SCD is important for the storage of fatty acids into hepatic lipids [94,106]. This effect was higher when the SO-diet was supplemented with cholesterol, provoking an increase of free cholesterol inclusion into very low-density lipoproteins particles as a consequence of the decrease in the hepatic storage capacity [94]. In addition, SO reduced the increased of plasma interleukin-6 (IL-6) levels in Otsuka Long–Evans Tokushima Fatty rats caused by obesity [52].

### 3.2. SA and Therapeutic Opportunities

**Figure 3:**
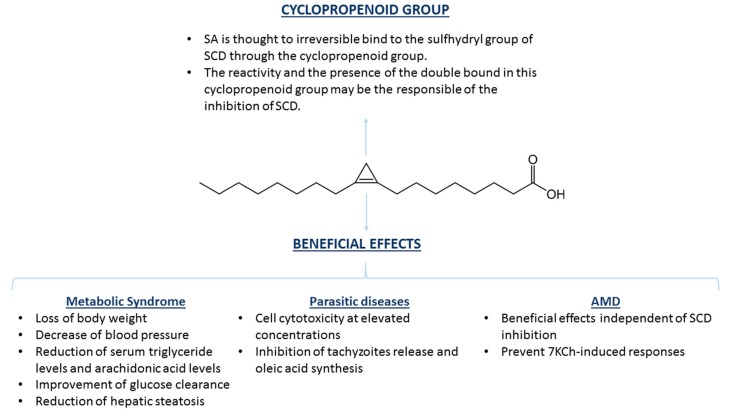
Chemical structure of SA and beneficial effects exerted in several pathologies. The cyclopropene group of SA has been suggested to be responsible for both binding and inhibition of SCD as a consequence of the reactivity of the double bond between C9 and C10. Inhibition of SCD by SA has been described as potentially therapeutic for several diseases, such as those related with metabolic syndrome and parasitic diseases. Positive effects of SA have also been shown in age-related macular degeneration (AMD), although the effects seem to be independent of SCD inhibition.

Many studies have described the role of increased SCD1 activity in different diseases [50,52,55,95], especially in those associated with lipid metabolism and metabolic syndrome [50,52,94]. Metabolic syndrome is defined as a group of at least three risk factors including obesity and increasing levels of triglycerides, lipoproteins (both low-density lipoproteins and high-density lipoproteins), and blood pressure, among other disorders, which increase the risk of suffering several cardiovascular diseases and type II diabetes mellitus [107]. In spontaneously hypertensive rats, administration of SO with or without anhydrous milk fat (which is rich in SFA, shown to have several beneficial effects) resulted in a decrease of blood pressure until normotensive values and in a loss of body weight (Figure 3). The positive effects correlated with a reduction in insulin concentrations. In addition, there was a significant decrease in the levels of serum triglycerides and fat weight when SO was administrated alone. Moreover, SO reduced the level of arachidonic acid in the membrane of their hepatocytes, which has been suggested to be a consequence of a decrease in the synthesis of eicosanoids derived from arachidonic acid, which has proinflammatory properties, and highlights the therapeutic potential of SA (Figure 3) [50]. Inhibition of SCD1 by SA as a treatment for obesity has also been studied in Otsuka Long–Evans Tokushima Fatty rats [52]. In this case, SO had no effect on body weight or total body composition, as it occurred in Zucker rats [51] However, glucose clearance had a 50% improvement, hepatic steatosis was reduced, and an amelioration of lipid profile was observed. However, insulin response did not change in the treated rats, which represents a limitation of the study [52].

A recent report showed the protective effect of SA, as well as several methyl and methoxy analogues of SA, against the parasite *Toxoplasma gondii*. SA inhibited the replication of intracellular tachyzoites, as well as its propagation, reducing the number of ruptured cells by the parasites. The study showed that SA is effective in inhibiting *Toxoplasma gondii* growth in vitro, suggesting that targeting SCD could be effective for the treatment of toxoplasmosis (Figure 3) [55]. Previous studies have also described the protective role of SA against other diseases caused by microorganisms. SA and its methyl sterculate derivatives prevented the synthesis of oleic acid in *Plasmodium falciparum*, the parasite responsible for the most severe form of malaria. This is hypothesized to provoke an imbalance between saturated and unsaturated lipids that seriously alters membrane fluidity and cellular traffic. A lack of oleic acid also hinders the formation of other lipids necessary for cell transduction and other important metabolic processes causing the inhibition of *Plasmodium falciparum* growth [54]. In *Mycobacterium tuberculosis*, SA inhibited the stearoyl-CoA desaturase DESA3, the enzyme responsible for oleic acid synthesis in this microorganism, showing a possible antituberculosis effect of SA since this enzyme is the target of thiourea isoxyl, which is an effective drug against this disease [56].

A recent study in Zucker rats, an animal model that presents obesity, metabolic dysfunction, and innate depression-like behaviors, showed that administration of SO had an anxiolytic-like effect by reducing the exploration latency and also improved the alterations in locomotor activity [51].

One of the most recent breakthroughs, with respect to SA, has been the discovery of its therapeutic potential against degenerative diseases [57,58]. SA could significantly counteract the inflammatory and cytotoxic responses caused by 7-ketocholesterol (7KCh), both in vivo and in vitro. 7KCh is an oxysterol that has been related to several degenerative diseases, including AMD [57,58,108], atherosclerosis [109,110,111,112], Parkinson´s [113], and Alzheimer´s disease [114,115,116]. A low concentrations of SA has been shown to protect ARPE-19 cells from 7KCh-induced cell death, as well as lessen the expression of several inflammatory cytokines, such as IL-1β, IL-6, IL-8 and vascular endothelial growth factor (VEGF) [117,118,119]. In addition, it reduced the expression of some ER stress markers, such as C/EBP homologous protein (CHOP) and glucose-regulated protein, 78KDa (GRP78). Other fatty-acids such as docosahexaenoic (DHA) or α-dihydrosterculic (DHSA) acid also showed a slightly protective effect, but they needed much higher concentrations to reach this effect. It was hypothesized that the cyclopropene group in the SA structure could be critical to its pharmacological properties, because this group is also present in the structure of DHSA, which partially protected the cells from death, but it does not appear in the structure of stearic acid, which did not show any protective effect [57]. SA also showed a protective effect in vivo in a laser-injury rat model, a suitable model for the study of angiogenesis where a laser burn induces choroidal neovascularization (CVN) and 7KCh is produced. Administration of SA via eye drops suppressed CNV volume by up to 67% [57].

Nowadays, it is not completely clear what the mechanisms are by which SA exert all its actions. For instance, it has been widely reported that it is a relatively potent inhibitor of SCD (IC_50_ = 0.9 uM). However, SA is also able to mediate anti-inflammatory and protective effects in the retina independently of SCD (AP, RP, APS, IML, in preparation) by a mechanism that could also involve the toll-like receptor 4 (TLR4) receptor and intracellular kinases [58] and could be of interest in other retinal pathologies [120,121].

## 4. Conclusions and Future Perspectives

Since 2005, when the first small molecule inhibitor of SCD1 was reported, numerous investigations have been carried out on the potential use of SCD1 inhibitors as new therapies for various diseases. Several active inhibitors with pharmacological activities in relevant animal models have been patented and reached human clinical phases. In addition to metabolic disorders such as diabetes and obesity, other therapeutic areas, for example, cancer, liver, and skin diseases have been actively pursued. However, the clinical utility of SCD1 inhibitors has faced some limitations due to the adverse effects of systemic administration and the mild therapeutic effects shown on some pathologies. Among the SCD inhibitors identified so far, SA is a natural compound with interesting properties. On the one hand, its ability to inhibit SCD would allow its use as a coadjuvant of several pathologies mentioned above. On the other hand, it seems to exert additional effects independent of its SCD inhibitory properties, which is particularly interesting in the treatment of retinal diseases such as AMD because the administration could be performed via eye drops which would greatly reduce the side effects that can be elicited by systemic administration.

## Figures and Tables

**Table 1:** SCD inhibitors used in the literature and related pathologies.

Inhibitor	Pathology	Tissue/Organ	Dose	Effect	References
A939572 (Bristol-Meyer Squibb)	Alopecia, Hypoplasia of meibomiam and sebaceous glands	Skin	3–60 mg/Kg	Sebaceous gland atrophy, reduction of lipid content	[14]
	Cancer	Pharynx Stomach Kidney Thyroid	19 nM 100 mg/Kg 6–65 nM 5–100 nM	Cell growth inhibition, cell death	[38,39,40]
		Cancer stem cells, mouse embryos Ovarian cancer Stem cells	75–100 nM 5 μM	Induce cell death thought ER stress, UPR Induce ferroptosis and apoptotic ceramides	[17,41]
CAY10566 (Cayman Chemical)	Cancer	Liver Ovary Colon	7–8 nM, 5 μM 1 μM	Cell growth inhibition, decrease in the oleic content, alterations in autophagy	[14,42,43,44]
		Cancer stem cells	75 nM 1–5 μM	Reduce cell viability Reduce NFκB signaling Induce ferroptosis and apoptotic ceramides	[17,41,42]
PluriSIn#1	Cancer	Cancer stem cells Cancer cell lines, Fibroblast, mouse embryos	20 μM	Induce cell death thought ER stress, UPR, and ROS/NOS	[41]
MF-438	Cancer	Thyroid Lung	2–5 nM	Cell growth inhibition, cell death, decrease of ALDH1A levels	[14]
		Lung cancer stem Cells Ovarian cancer Stem cells	0.007–50 μM 1 μM	Induce cycle arrest, apoptosis, RE stress, and autophagy Induce ferroptosis and apoptotic ceramides	[17,45]
MK-8245 (Merk Frosst)	Type II diabetes, dyslipidemia, obesity	Liver	20–60 mg/Kg	Antidyslipidemic Antidiabetic	[46]
SCDi I/II	Cancer	Breast and prostate cancer cell lines	0.001 nM–100 μM	Cytochrome C dependent apoptosis and tumor growth inhibition	[47]
CVT-11127	Cancer	Lung cancer cells	1 μM	Inhibit cell cycle and induce cell apoptosis	[48]
XEN103 (Novartis)	Acne	Skin	2–14 nM	Sebaceous gland atrophy Blockage of SCD1 transcription induced by androgens Phosphatidylcholine decrease of monounsaturated acyl chains	[14,49]
Sterculic Acid	Metabolic syndrome	Adipose tissue Liver	Diets supplemented with 0.4%–1.3% of SO	Blood pressure decrease, loss of body weight, decrease of serum triglyceride levels, decrease of arachidonic acid levels, improvement of glucose clearance, reduction of hepatic steatosis	[50,51,52,53]
	Parasitic diseases (toxoplasmosis, malaria, and tuberculosis)	Small intestine Erythrocytes Lungs	870 μM–1 mM, 10–100 μg/mL	Cell cytotoxicity at high concentrations, inhibition of tachyzoites release, inhibition of oleic acid synthesis	[54,55,56]
	Age-related macular degeneration	Retina	1–10 μM	Counteracts the inflammatory and cytotoxic effects of 7-ketocholesterol, but seems to be independent from its capacity to inhibit SCD1	[57,58]
